# Tn*6603*, a Carrier of Tn*5053* Family Transposons, Occurs in the Chromosome and in a Genomic Island of *Pseudomonas aeruginosa* Clinical Strains

**DOI:** 10.3390/microorganisms8121997

**Published:** 2020-12-15

**Authors:** Vaheesan Rajabal, Vilma A. Stanisich, Steve Petrovski

**Affiliations:** Department of Physiology, Anatomy and Microbiology, La Trobe University, Bundoora, VIC 3086, Australia; vaheesan.r@gmail.com (V.R.); vstanisich@bigpond.com (V.A.S.)

**Keywords:** Tn*5053* family transposons, Tn*6603*, clinical *Pseudomonas aeruginosa*, genomic island PAGI-5v, class 1 integrons, nested transposons, defective transposons, ICE family

## Abstract

Transposons of the *Pseudomonas* *aeruginosa* accessory gene pool contribute to phenotype and to genome plasticity. We studied local *P. aeruginosa* strains to ascertain the encroachment of *mer*-type *res* site hunter transposons into clinical settings and their associations with other functional modules. Five different Tn*5053* family transposons were detected, all chromosomal. Some were solitary elements; one was in *res* of Tn*1013^#^*, a relative of a reported carrier of *int*-type *res* site hunters (class 1 integrons), but most were in *res* of Tn*6603*, a new Tn*501*-related transposon of unknown phenotype. Most of the Tn*6603*::Tn elements, and some Tn*6603* and Tn*6603*::Tn elements found in GenBank sequences, were at identical sites in an hypothetical gene of *P. aeruginosa* genomic island PAGI-5v. The island in clonally differing strains was at either of two tRNA^Lys^ loci, suggesting lateral transfer to these sites. This observation is consistent with the membership of the prototype PAGI-5 island to the ICE family of mobile genetic elements. Additionally, the *res* site hunters in the nested transposons occupied different positions in the Tn*6603* carrier. This suggested independent insertion events on five occasions at least. Tn*5053* family members that were *mer*-/*tni*-defective were found in Tn*6603*- and Tn*501*-like carriers in GenBank sequences of non-clinical *Pseudomonas* spp. The transposition events in these cases presumably utilized *tni* functions in trans, as can occur with class 1 integrons. We suggest that in the clinical context, *P. aeruginosa* strains that carry Tn*6603* alone or in PAGI-5v can serve to disseminate functional *res* site hunters; these in turn can provide the requisite *trans*-acting *tni* functions to assist in the dissemination of class 1 integrons, and hence of their associated antibiotic resistance determinants.

## 1. Introduction

*Pseudomonas aeruginosa* is a metabolically versatile bacterium that inhabits many diverse ecological niches and is also a significant opportunistic nosocomial pathogen in humans [1,2]. The treatment of infections may be severely compromised by the organism’s broad intrinsic resistance to antimicrobial agents and its ability to acquire resistance through horizontal gene transfer processes [1]. The inherited genes are frequently located within mobile genetic elements (MGEs), combinations of which contribute to the accessory gene pool of individual strains and aid in niche adaptation [3].

The rich repertoire of MGEs in *P. aeruginosa* includes plasmids, transposons (Tn), genomic islands (GIs), integrative and conjugative elements (ICEs), and integrons (In), which are often present in strains that exhibit combined resistance to mercury(II) (*mer*) and to antibiotics. The former phenotype is associated with *mer* transposons [4,5] or independent *mer* modules, for example in ICEs and GIs [6,7,8], or in combination with antibiotic-resistance genes, such as occurs in the prevalent IncP-2 plasmids and the ubiquitous IncP-1 plasmids that occur widely in Gram-negative bacteria [9,10,11,12]. Some IncP-1 plasmids have antibiotic-resistant genes that are fixed in a conserved plasmid backbone, which is often interrupted by accessory MGEs [11]. Tn*21*/Tn*501*-like elements predominate in the *trfA-oriV* region and include relatively simple structures (e.g., Tn*1696*, a *mer* transposon carrying In4; [13]) and complex arrangements (e.g., Tn*511*, with components from five MGEs; [14]). On the other hand, Tn*5053*/Tn*402*-like elements predominate in the *parA-res_IncP_* region owing to a specific targeting mechanism, as described below. The focus of the present study is the Tn*5053* family of *mer*-type transposons. This group has the capacity to aid in the spread of class 1 integrons [15,16], which are prominent members of the Tn*402* family and encode a multiplicity of antibiotic-resistant gene cassettes [17,18].

**Figure 1:**
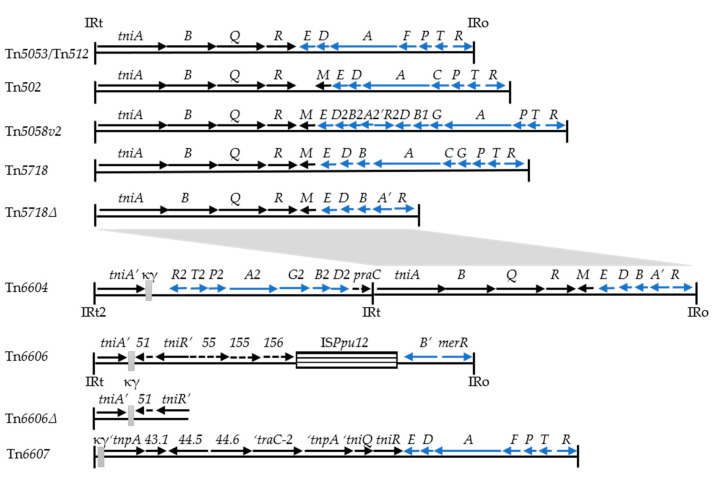
Structures of Tn*5053* family transposons. The transposition genes (*tni* and *tnp*), the mercury resistance genes(*mer*) and hypothetical genes (*orfs*) are represented as named or numbered arrows; remnant genes are indicated by prime notation (e.g., *tniA’*) and phosphoribosyl-AMP cyclohydrolase by *praC*. The positions of IRs (25 bp) are shown, and inserted MGEs are represented as boxes. Identity (95%) between Tn*5718Δ* and Tn*6604* is indicated by the grey box. Putative products of the *orf*s are described in Figure 5a and by Haines et al. [24] for Tn*6607*. The Figure was generated from the following GenBank entries: Tn*5053* (L40585); Tn*502* (EU306743); Tn*512* (EU306744); Tn*5718* (AJ304453); Tn*5718Δ* (AM261760); Tn*6606* (AJ344068); Tn*6606Δ* (KF743817); Tn*6607* (previously unnamed; AM157767).

Tn*5053* and Tn*402*, the prototype transposons of the *res* site hunter family [19], probably share a common ancestor; their transposition genes are related (*tniABQ-res-tniR*; 83% identity) and the elements are bordered by characteristic 25 bp inverted repeat termini (IRo and IRt) [20,21,22]. Less than 10 distinctive Tn*5053* family members are known, based on the gene organization and sequence diversity of complete elements, although there is evidence for others ([22,23,24]; Figure 1). All have an exogenously captured *mer* module and most are functional transposons. They have been isolated from bacterial species in diverse environments, including ancient sediments, with Tn*5053* being especially widespread [23,25,26]. There are few examples from the clinical setting [22,27]. Tn*402* and some close relatives are also functional transposons. Their *tni* module has a captured *int* module (i.e., integrase gene, *intI1*, and an *attI1* recombination site; [16]) located close to the site of the *mer* module found in the Tn*5053* family. Functional Tn*402*-type elements (self-mobile integrons) do not appear to be common in the clinical setting [7,28]. Instead, many members of the Tn*402* family are defective transposons (class 1 integrons) derived from a Tn*402* ancestral element that lost *tni* genes during evolution [21,29,30,31]. A third Tn*5053*/Tn*402*-related family is defined by Tn*6048*, a multi-metal response transposon from *Cupriavidus* and *Variovorax* [32,33].

Information on the transposition process comes from studies on Tn*5053*, Tn*502*, and Tn*402* [19,34,35,36,37]. These move by replicative transposition, preferentially inserting at a *res* site (hence the “*res*-site hunter” epithet) by using the associated target site resolvase and the *trans*-acting TniABQ proteins to form a cointegrate. This is resolved by *cis*-acting components (*res-*TniR) aided by the target site resolvase. The Tn is inserted in a preferred orientation (IRo nearest the target site resolvase gene) and is flanked by 5 bp direct repeats (DR). Not all *res* sites serve as suitable targets, and transposition to non-*res* sites is inefficient [19,37]. Class 1 integrons at their locations in *res* sites of various MGEs also exhibit key indicators of Tn*5053*/Tn*402*-type transposition, fostering recognition that they are defective transposons [21,29]. Indeed, such linkage to MGEs has promoted the spread of class 1 integrons among Gram-negative bacteria, including instances of Tn-In elements in ICEs and GIs [15,17,38].

Mechanisms that mediate the lateral movement of class 1 integrons, independent of an MGE, remain a moot point. Theoretical considerations suggest that integrons that retain intact IRs can be mobilized if a complete *tni* module is provided in trans [21,29]. Experimental evidence of such events is the movement of In0 and In2—these have retained only *tniA*, yet they transpose to a plasmid site (*res_IncP_*) when assisted by *tni* genes from Tn*502* in trans [37]. The efficiency of the resolution, which is effected by the host *recA* system and the target-site resolvase, is normal, corresponding to that seen with a fully functional element (e.g., Tn*502*). The movement of Tn*2521* (=In33) to *res_IncP_* has also been observed and is suspected to have involved *tni*-assisted transposition by another Tn [39,40]. Similar assisted transposition events explain the occurrence of class 1 integrons in *res* sites of different plasmids and transposons, including *mer* and non-*mer* transposons (see [41] for references). Experimentally, Tn*5053* targets some of the same *res* sites in transposons, as well as additional sites [19].

This study focusses on the Tn*5053* family, which is one of three transposon groups that have contributed to the worldwide dissemination of *mer* genes in environmental bacteria [25]. Additionally, members of the Tn*21* family are broadly distributed in clinical settings [5,26], but whether this is the case for the Tn*5053* family is largely unexplored [22,27]. This aspect of the biology of the Tn*5053* family warrants study, especially as class 1 integrons have on independent occasions inserted into various *mer* transposons, possibly in the clinical environment [27,41]. These linkage events, as described above, require the in trans supply of functional *tni* genes; the Tn*5053* family is a possible source. We did indeed detect members of this family among locally isolated clinical strains of *P. aeruginosa.* Unexpectedly, most were embedded in a new carrier transposon, Tn*6603*, which is also often linked to the same GI. Tn*6603*, regardless of whether or not it is a part of the GI, has the capacity to attract *res* site hunters, including class 1 integrons, and to associate with MGEs. As such, Tn*6603* can contribute to the ongoing adaption of *P. aeruginosa* to antimicrobials in the clinical setting, and also to the catabolic activities of *Pseudomonas* spp. in non-clinical environments.

## 2. Materials and Methods

### 2.1. Bacterial Strains, Plasmids and Growth Media

The *Escherichia coli* K-12 derivatives used were DH5α (*recA1* Res^−^ Mod^+^ Nal^r^) [42] and LT101 (*recA13* Res^−^ Mod^−^ Str^r^ Rif^r^ auxotroph) [43]. The *Pseudomonas aeruginosa* strains were PAO9503 (Str^r^ auxotroph) [44] and 191 wild type strains from clinical sources. The properties of the plasmids used are listed in Table 1, and detailed characteristics of 12 of the clinical strains are shown in Table 2. Nutrient agar (NA) and nutrient broth (NB) have been described in [43]. For selection of *E. coli* strains (or *P. aeruginosa*, as indicated), antimicrobials were added to NA at the following final concentrations (µg × mL^−1^): carbenicillin (Cb) at 100 or 250 (for *P. aeruginosa*); chloramphenicol (Cm) at 10; mercuric chloride (Hg) at 15 (for both *E. coli* and *P. aeruginosa*); nalidixic acid (Nal) at 8; streptomycin (Sm) at 100 (for both *E. coli* and *P*. *aeruginosa*); tetracycline hydrochloride (Tc) at 10 or 80 (for *P. aeruginosa*). Carbenicillin was used to select strains carrying ampicillin (Ap)-resistant plasmids. Media containing these various agents are presented in an abbreviated form. For example NA.Sm.Hg indicates nutrient agar with added streptomycin and mercuric chloride.

### 2.2. Transposons and Plasmid Constructions

The transposons and nested transposons named in Table 2 were sequenced, as was the new transposon Tn*6603* isolated from *P. aeruginosa* PA65. The naming of new transposons in this work (i.e., Tn*6603*, Tn*6604*, Tn*6606*, and Tn*6607*) follows the recommendations of the transposon registry (https://transposon.lstmed.ac.uk) and is based on evidence of transpositional insertion and complete nucleotide sequence. The plasmids used are listed in Table 1, except for additional derivatives of pUB307 and pUB1601, which are listed in Table 2. Details of plasmid constructions involving DNA techniques are given in the footnotes to Table 1.

### 2.3. Conjugation, Transposition Assays, and Analysis of Transconjugants

Late-exponential-phase cultures in NB were used for conjugation experiments conducted using the quantitative filter method [43]. Experiments using independent cultures were performed three times and the average transfer frequency (transconjugants per donor) was calculated.

Detection of *res* site hunter transposons: *P. aeruginosa* strains were grown at 43 °C to induce a restriction-deficient phenotype [45] prior to conjugation with DH5α-donors carrying either pUB307 or pUB1601. Transconjugants were recovered on NA.Hg.Tc. Representative transconjugants were subjected to three cycles of subculture and subsequently used as donors in conjugations with PAO9503 grown at 43 °C. Transconjugants were recovered on NA.Sm.Hg. Plasmid DNA extracted from purified transconjugants was sequenced to determine the presence of pUB307 or pUB1601 derivatives carrying either *res* site hunter or nested transposons.

Characterization of Tn*6603c*: (a) Transposition was studied using a DH5α derivative carrying pSK::Tn*6603c* and conjugally introduced pUB307 (or pUB1601) (selection was on NA.Nal.Tc). A mixture of the transconjugants was subcultured three times on NA.Nal.Tc.Cm and a donor culture prepared in 10 mL NB for use in an outcross to LT101. The number of transconjugants isolated on NA.Rif.Cm enabled determination of the transposition frequency. Plasmid DNA extracted from transconjugants from separate experiments was sequenced to determine the location of the inserted Tn*6603c*. (b) Conductional transfer was studied using a DH5α derivative carrying pBR322 and conjugally introduced pSP116 (or pSP145) (selection was on NA.Km). As in (a), transconjugants were subcultured on NA.Km.Cb for subsequent use in an outcross to LT101. The number of transconjugants isolated on NA.Rif.Cb enabled determination of Tn*6603c*-mediated conductional transfer relative to that by Tn*501*. (c) Targeting of Tn*502* to *res_6603c_* and *res_IncP_* was studied using a DH5α derivative carrying pSP114 (or pVS76) and conjugally introduced pSP144 (selection was on NA.Km). As in (a), transconjugants were subcultured on NA.Km.Tc for subsequent use in an outcross to LT101. Transconjugants were isolated on NA.Rif.Tc. Plasmid DNA extracted from transconjugants from separate experiments was transformed into DH5α, with selection on NA.Tc. and subsequent screening to identify Hg^r^ Km^s^ transformants. The resolved pSP114::Tn*502* (or pVS76::Tn*502*) plasmids in these transformants were isolated and sequenced to determine the location of the inserted Tn*502*.

### 2.4. DNA Techniques, Nextgen Sequencing, and Analysis

Cloning, DNA manipulations, and Southern (colony lift) hybridizations were performed as described previously [46]. PCR amplification involved the use of iProof™ high fidelity DNA polymerase (Bio-Rad, Gladesville, New South Wales, Australia). Reaction mixtures were placed in a thermal cycler for 35 cycles under the following conditions: 98 °C, 2 min (first cycle only); 98 °C, 30 s; 72 °C, 15 s/kb; 72 °C, 10 min (last cycle only). The reaction products were detected using agarose gel electrophoresis and staining with Invitrogen SYBR safe (Thermo Fisher Scientific, Scoresby, Victoria, Australia). The digoxigenin-labelled *tniA_502_* probe used in hybridization was prepared from the PCR products obtained using primers VR1 (5′-GTGCGAGTGATGACGGTTG) and VR2 (5′-GTGCAGATTGATCACACGG), with pVS983 DNA as the template. Primers VR49 (5′-CCCTTCGTGCGGATAGTCAG) and VR50 (5′-TTCCCGAAGGCGTTTCTTGA) as well as primers VR51 (5′-AGCTCGACTTGGGTTCCATC) and VR52 (5′-CAGATCAGGTCGCAGCAAAG), which are specific for the *orf*_6603_ and *res_6603_* regions, respectively, were used to screen for the presence of Tn*6603* in *P. aeruginosa* clinical strains. In addition, to identify the presence of Tn*6603* or Tn*6603*::Tn nested elements in the chromosomal *orfX* gene of the clinical strains, primers VR131 (5′-ATGAAGCAGACCTTCGAATA) and VR135 (5′-CGCCATTGTGTTGTGGAACA) as well as primers VR132 (5′-TTATGAGGTTGCGCGCTTG) and VR136 (5′-GCCATCACGCTCCAGTAACT) were used. These primer-pairs amplify the *orfX-tnpA_6603_* (304 bp) and *orf41_6603_-orfX* (520 bp) junction fragments, respectively. Primer pairs used for strain construction (VR56/VR67 and VR75/VR76) are shown in Table 1. Plasmid DNA was isolated from 10 mL overnight NB cultures. DNA from *P. aeruginosa* PA65 and strains listed in Table 2 was obtained using the Wizard genomic extraction kit (Promega, Alexandria, NSW, Australia); the QIAprep Spin Miniprep Kit (Qiagen, Chadstone, VIC, Australia) was used to screen for plasmid DNA in clinical strains, or to isolate pUB307/pUB1601 plasmids carrying *res* site hunter or nested transposons, both according to the manufacturers’ instructions. DNA libraries were prepared for sequencing using the Nextera^®^ XT V2 DNA library preparation kit (Illumina, San Diego, CA, USA) according to the manufacturer’s instructions. DNA sequencing was performed using a 300 bp paired-end reads Miseq^®^ V3 reagent kit (300 cycles) (Illumina, San Diego, CA, USA) on an Illumina MiSeq^®^ (Illumina, San Diego, CA, USA). De novo assembly of the sequenced reads were conducted using CLC Workbench (version 9.5.1, CLC bio, a QIAGEN Company, Prismet, Aarhus, Denmark) or Unicycler [47]. Gene annotation was manually performed using the Glimmer Prediction tool on Geneious Prime^®^ (version 2019.0.3, Biomatters, Ltd., Auckland, New Zealand). Gene sequences were confirmed using BLAST NCBI.

### 2.5. Nucleotide Sequence Accession Numbers

Nucleotide sequences accession numbers of mobile elements deposited in GenBank are as follows: Tn*6603* (MT043136), Tn*6604* (MT043137), Tn*5058v2* (MT043138), and Tn*1013^#^* (MT043138). The accession numbers of sequences used for comparative studies are: Tn*1013* (AM261760) and RP1 (BN000925). Others are listed in figure descriptions. Accession numbers of GenBank sequences containing Tn*6603* or Tn*6603* with inserted *res* site hunter transposons are shown in Table S1.

## 3. Results

### 3.1. Isolation of Res Site Hunter Transposons, Including the New Element Tn6604, from Clinical Strains of P. aeruginosa

We studied 191 *P. aeruginosa* strains obtained from hospitals in Melbourne in the period 1988–2015. Fourteen strains were found to hybridize with a probe (*tniA_502_*) of the Tn*502* transposase gene, of which 12 were mercury-resistant (Hg^r^). Each of the latter group carried a presumptive Tn*502*-related *mer* transposon, probably chromosomal, as no plasmid DNA was detected, nor was Hg(II) resistance transferable to a recipient strain (PAO9503) by conjugation (Table 2 and footnote a).

To recover transposons, plasmid pUB307 was used as a “bait”, as it carries a target site (*parA-res_IncP_*) favored by *res* site hunters (Table 1) [19,21,37,53]. When pUB307 was passaged through the 12 strains and then to strain PAO9503, all yielded pUB307-Hg^r^ plasmids (at ca 10^−2^–10^−5^/donor; Table 2). One plasmid from each strain, when sequenced, was found to have a *res* site hunter transposon inserted in the *res_IncP_* sequence (Table 2 and footnote g) and oriented with the IRo-end adjacent to *parA*. Ten of the transposons were identical (99–100%) to prototype elements: namely, to Tn*502* [22], Tn*512* [22], and Tn*5053* [21] (Table 2). Another transposon, Tn*5058v2*, was identical to the prototype Tn*5058* [26], except for having an extra gene remnant (*merA2′*, 229 bp) (Figure 1). All eleven transposons had a simple structure consisting of *mer* genes and *tni* genes bounded by 25 bp IRs (Figure 1).

**Figure 2:**
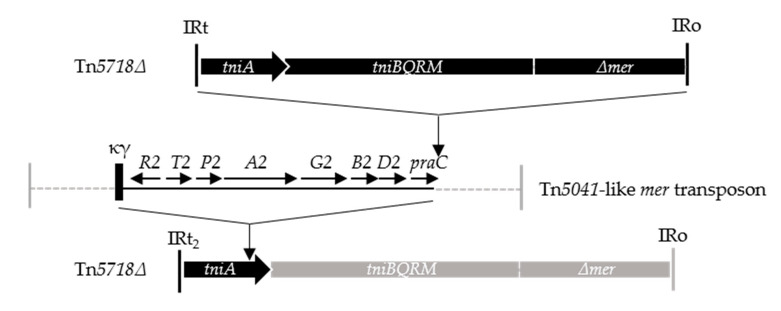
Hypothetical genesis of Tn*6604*. Three events are envisaged (see Figure 1 and text). A Tn*5718Δ-*like element was disrupted by a Tn*5041*-like *mer* transposon; subsequently, κϒ-mediated deletion fused κϒ-*merR*-*praC* sequences to the IRt2-*tniA*’ remnant. A Tn*5718Δ*-like element inserted into the Tn*5041* entity to the right of *praC*. Tn*6604*, bordered by IRt2 and IRo, is shown in dark shading; grey shading represents regions absent from Tn*6604*. Here, *Δmer* represents an incomplete *mer* module (see Figure 1).

In contrast, the last transposon, named Tn*6604* (14,858 bp), consisted of three discrete parts (Figure 1), each probably from a different interacting element (Figure 2). The right arm of Tn*6604* (7176 bp) is 95% identical to Tn*5718Δ* detected in a *P. aeruginosa* IncP-1α plasmid [54]. Tn*5718Δ* has an incomplete *mer* module, but its *tni* module and terminal IRs are intact. The central portion of Tn*6604* (6399 bp) shares high identity (99%) with the chromosomal *mer* region of *P. putida* DLL-E4 (accession no. CP007620). The same *mer* genes occur in *mer2* variants of Tn*5041*, a Tn*3* family transposon detected in *P. putida* and *P. fluorescens* strains [55]. The *P. putida* DLL-E4-related sequences in Tn*6604* terminate in the miniature element κϒ (262 bp) [56]. The short left arm of Tn*6604* (1284 bp) is 99% identical to Tn*5718Δ*/Tn*5718* [54,57]. It consists of a *tniA* remnant (1141 bp), directly flanked by κϒ and by intergenic sequences adjoining the terminal IRt2 (Figure 1). Our ability to detect pUB307::Tn*6604* plasmids relied upon the expression of Tn*6604* transposition functions (IRt2, *tni* genes, IRo) and *mer2* resistance functions distributed across the three parts (Figure 2). The Tn*5718Δ*-like component of Tn*6604* is also presumably self-mobile, since it encodes the *tni* functions and its IRt1 is identical to IRt2 of Tn*6604* (Figure 1).

The various *mer*-type *res* site hunters identified here, and including the previously reported Tn*512* [22], are the first described instances in bacterial strains from Australia. This is despite their presence in clinical *P. aeruginosa* strains as early as 1988–1991 (Table 2). Their detection is significant because, to our knowledge, similar surveys of clinical localities elsewhere have not been reported.

### 3.2. Isolation of Res Site Hunter Transposons Nested with a Carrier Transposon

Our detection of *res* site hunter transposons (Table 2) relied on the established affinity of such elements for the plasmidal *parA-res_IncP_* region [34,35,37,53]. However, transposon-encoded *res* regions can also serve as suitable targets [19,41,58]. To detect nested associations of a *res* site hunter in *res* of a carrier transposon, we employed pUB1601 as the “bait” (Table 1). This plasmid lacks *parA-res_IncP_*, thereby limiting acquisition of a *res* site hunter [37], whilst allowing acquisition via a carrier element.

Plasmid pUB1601 was passaged through the 12 strains that harbored a *res* site hunter transposon, followed by outcrossing to PAO9503 to recover pUB1601-Hg^r^ recombinants. This strategy was successful from ten strains, although at lower frequencies (ca 10^−5^–10^−8^/donor) than in the experiments involving pUB307 (Table 2). A plasmid from each of the ten experiments was sequenced. Two (from strains AW57 and PA9) were pUB1601::Tn*502* plasmids. These represent examples of rare, random insertion events noted previously with Tn*502* [37] (Table 2, footnote h). In the remaining eight, the *res* site hunter (Tn*502*, Tn*5053*, Tn*5058v2*, or Tn*6604*) was inserted in one of two carrier transposons, forming a larger nested arrangement. The nested transposon was at different locations in pUB1601 (Table 2, footnote h).

**Figure 3:**
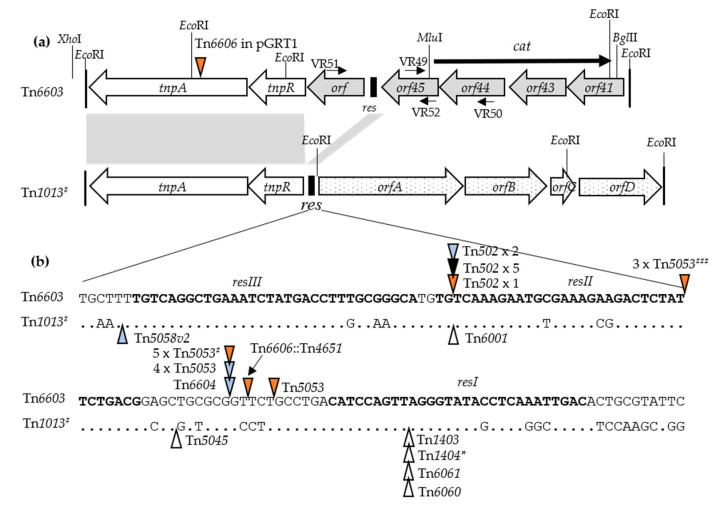
(**a**) Structures of Tn*6603* and Tn*1013^#^*: The *tnp* genes and *orfs* are represented as arrows and the *res* sites as black boxes. The positions of IRs (38 bp) are indicated by vertical lines; some include *Eco*RI sites. The *cat* gene in Tn*6603c* is shown overlying the *orfs* it replaces in Tn*6603*. Identity (92%) between the *tnp* regions is indicated by grey boxes. The locations of primers pairs (VR49/VR50 and VR51/VR52) used in PCRs, and of restriction enzyme sites used for strain construction, are shown. Putative products of the cargo genes are: Orf41, cupin transcriptional regulator; Orf43, TetR/AcrR family transcriptional regulator; Orf44, NAD(P)-dependent oxidoreductase; Orf45, osmotically induced detox protein; Orf, unknown; OrfA, sodium-independent anion transporter; OrfB, universal stress protein UspA; OrfC, molecular chaperone DnaK; OrfD, DNA binding protein (as annotated in accession no. NC_003350 and KY494864). (**b**) Sequence of the *res* region of Tn*6603* and Tn*1013^#^* and transposon insertion sites: The *resIII*, *resII*, and *resI* sequences are shown in bold. Periods represent nucleotides identical to those in Tn*6603*. Triangles represent insertion sites of *res* site hunter transposons. Color coding is as follows: Blue, transposons shown in Table 2. All are in *orfX*::Tn*6603* strains except for one Tn*502* and one Tn*5058v2* strain. Orange, transposons from GenBank sequences. All are in *orfX*::Tn*6603* strains except for four Tn*5053* strains (marked by ^#^). For Tn*6606* and Tn*6606*::Tn*4651,* see Figure 5. White, integrons from GenBank sequences. Black, laboratory-derived independent insertions (this study).

In the case of Tn*5058v2*, the carrier transposon was a close relative of Tn*1013* (accession no. AM261760). It was denoted Tn*1013^#^* for discussion purposes. Tn*1013^#^* (7875 bp) and Tn*1013* (7800 bp) share high identity (99%) across their *tnpAR-res* modules, which are related to transposons in the Tn*21*/Tn*501* family. Their associated cargo genes (*orfABCD*) are less similar (ca 94% identity) and the *orfC* of Tn*1013^#^* is longer by 75 bp. Putative functions have been ascribed to *orfABCD,* but no associated phenotypes are known (Figure 3). Tn*5058v2* was inserted in *res_1013#_* (near *resIII*; Figure 3) and was flanked by 5 bp DRs (TGAAT), indicating that the nested structure arose from a direct transposition event. The association between Tn*1013^#^* and Tn*5058v2* is the first description of a *mer*-type *res* site hunter in a Tn*1013*-like carrier. This contrasts with at least six descriptions of Tn*1013*-like carriers bearing class 1 integrons (i.e., Tn*1403* and Tn1404* [59], Tn*6001* [60], Tn*6060* [61] and Tn*6061* [62], all from clinical *P. aeruginosa* strains, and Tn*5045* [41], from a *Pseudomonas* permafrost strain). With one exception (Tn*6061*), the respective integrons are *tni*-defective, but retain their IR_25_ borders. All are inserted within *res_1013_* (Figure 3) and three (Tn*1403*, Tn1404*, and Tn*5045*) are flanked by 5 bp DRs. These features suggest that at least for the latter three cases, and probably for all, the integron transposed directly into Tn*1013* assisted by *tni* functions present in trans. Similar transposition of In0 and In2 has been demonstrated experimentally [37].

In the case of the nested associations involving Tn*502*, Tn*5053,* and Tn*6604*, a new carrier element was identified and named Tn*6603* (7315 bp) (Table 2, footnote i). Tn*6603*, as with Tn*1013*, is a member of the Tn*21*/Tn*501* family. However, the *tnp_6603_* module shares only 92% identity with the *tnp_1013_* module, while in Tn*6603*, *tnpR* is separated from *res* by a putative 465 bp *orf* of unknown function (Figure 3). Additionally, Tn*6603* contains a cargo of four genes, which are different from those in Tn*1013* and transcribed in the opposite direction. Genes similar to those in Tn*6603* (99% identity) occur in Tn*4653*, a catabolic transposon in *P. putida* plasmid pWW0 [63]. Putative functions have been ascribed to the genes (named *orf41-43-44-45*) but are not associated with a known phenotype (Figure 3). In each nested element, the *mer* transposon was inserted in *res_6603_* (in *resII* for Tn*502* and the same site between *resI* and *resII* for Tn*5053* and Tn*6604*; Figure 3) and was flanked by 5 bp DRs (ATGTG and GCGCG, respectively). This indicates that the transposon inserted into the Tn*6603* carrier on at least three separate occasions. Figure 3 shows the sequences of *res_6603_* and *res_1013#_* and the distribution of insertion sites for the *mer* transposons reported here and the integrons reported by others. Most insertions cluster in the *resI-resII* region, whereas Tn*502* insertions are at a single, more distant *resII* site, while Tn*5058v2* is near *resIII*.

### 3.3. Isolation and Characterization of the Carrier Transposon Tn6603

Tn*6603* was linked with Tn*502*, Tn*5053,* or Tn*6604* in seven of the surveyed strains (Table 2). To detect instances of the “unloaded” Tn*6603*, the strain collection was subjected to PCR analysis using appropriate primers pairs (*orf*-specific VR49/VR50; *res_6603_*-specific VR51/VR52; Figure 3). Six of 191 strains, all Hg(II)-sensitive, yielded products in the expected size ranges (i.e., 871 bp and 1095 bp, respectively). One strain (*P. aeruginosa* PA65) was sequenced, revealing a single chromosomal copy of Tn*6603* (7315 bp) flanked by 5 bp DRs (ACCAA). This suggested that Tn*6603* had inserted at that site by direct transposition. Tn*6603* was PCR-amplified (using primers VR56/VR67) and the product was cloned into pBluescript SK+, forming pSP111. Further study of Tn*6603* was conducted using Tn*6603c* (9474 bp), which has a chloramphenicol resistance gene replacing *orf41*–*44* (Table 1; Figure 3).

Experimentally, Tn*6603c* was found to relocate from pBluescript SK+ to pUB1601 (and to pUB307) at frequencies of ca 1.2 × 10^−3^/donor in the absence of host RecA function (see Materials and Methods). Only resolved products were detected (e.g., pUB1601::Tn*6603c* plasmids) and Tn*6603c* was inserted at different sites in the plasmid backbone (near *oriV*, *korC*, *klaC,* and *klaB*), in each instance flanked by 5 bp DRs. Thus, Tn*6603c* is a functional transposon with low-level target specificity. Conductional studies to identify cointegrate formation were also performed, comparing Tn*6603c* with Tn*501*, which moves by replicative transposition [5]. Transfer of pBR322 (which lacks an *oriT*) was mediated both by pUB1601*tet*::Tn*6603c* and pUB1601*tet*::Tn*501* (at 2.0 × 10^−4^/donor and 3.5 × 10^−3^/donor, respectively) (see Materials and Methods). These findings demonstrate that the transposition of Tn*6603c* involves a cointegrate intermediate.

Lastly, we used Tn*502* to affirm that the various nested combinations in the clinical strains (Table 2) arose by transpositional targeting to the *res_6603_* site. Plasmid pUB1601*tet*::Tn*502* served as the transpositional donor in a strain also carrying either pBR322-*res_6603_* (i.e., with the *tnpR-res_6603_* region) or pBR322-*res_IncP_* (i.e., with the *parA-res_IncP_* region). Conductional transfer of pBR322-*res_6603_* was detected at a frequency of 2.1 × 10^−6^/donor compared to 4.4 × 10^−5^/donor for pBR322-*res_IncP_* (see Materials and Methods). The resolved pBR322 components from independent experiments were isolated and sequenced. In the five plasmids tested, Tn*502* occurred at the same site in the *resII_6603_* sequence, flanked by 5 bp DRs (ATGTG); the same fusion site also occurred in the Tn*6603*::Tn*502* transposons from strains RH19 and AW32 (Table 2; Figure 3). Tn*502* also targeted the *resII_IncP_* sequence at the same relative position as in *resII_6603_*, but was flanked by different DRs (TTAAA) (three plasmids tested). These data confirm that the *res_6603_* region can serve as the target for *res* site hunter transposons and is favored relative to other sites in Tn*6603*.

### 3.4. Distribution of Tn6603 and Tn6603-Nested Transposons and Their Association with Genomic Island PAGI-5v

We detected Tn*6603* in six local *P. aeruginosa* clinical strains and Tn*6603*::Tn-nested elements in seven others (Table 2). A search of the GenBank database revealed additional homologues of Tn*6603*, none less than 99% identity, and none named or annotated as transposons. These were 14 examples of Tn*6603*, one Tn*6603*::Tn*502,* and nine Tn*6603*::Tn*5053* (Table S1). All were chromosomally located in *P. aeruginosa* strains, except for plasmid-borne instances in *P. aeruginosa*, *P. fluorescens,* and *P. taiwanensis* strains (1 example each). The strains were from diverse locations (e.g., UK, Europe, USA, Mexico, Hong Kong, and Kuwait), all but one isolated since 2010, and many (12/24) were clinical isolates. Therefore, Tn*6603* in its native and enlarged forms is widely distributed, with *P. aeruginosa* as the predominant host.

With two exceptions, the various Tn*6603*-entities were flanked by 5 bp DRs and occupied nine disparate locations, consistent with the transpositional behavior observed with Tn*6603c*. One site was nonetheless overrepresented, with all the insertions (five of Tn*6603*, one of Tn*6603*::Tn*502,* and five of Tn*6603*::Tn*5053*) at the same chromosomal position—nt61 of a hypothetical *orf*, here named *orfX*. Examination of the *orfX* region in our local strains (using primer pairs VR132/VR136 and VR131/VR135; see Materials and Methods) showed that six of the nested elements were also at position nt61 (i.e., Tn*6603* nested with Tn*502*, Tn*5053* or Tn*6604*; Table 2). The relationship between all of these strains most likely arises from a single transposition event that formed a *P. aeruginosa orfX*::Tn*6603* common ancestor. The ancestral strain or derivatives subsequently inherited Tn*502* or Tn*6604* on at least one occasion each, or Tn*5053* on at least three separate occasions, based on the different locations of the *mer* transposons in *res_6603_* when the GenBank sequences are included (Figure 3).

**Figure 4:**
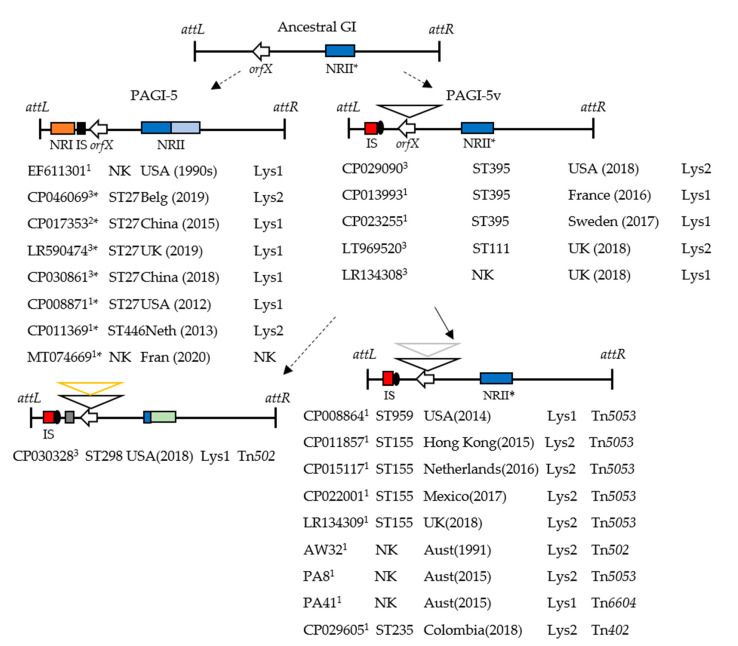
Hypothetical genesis of PAGI-5-related genomic islands in *P. aeruginosa*. The features shown for PAGI-5 ([6]; EF611301) and for PAGI-5v-lineages are shared by the strains listed under each figure according to accession number, sequence type (ST) (NK = not known), country of origin, date of isolation, location of the GI relative to the tRNA^Lys^ gene copies (i.e., Lys1 = *ca* 1.06 Mbp or Lys2 = *ca* 5.08 Mbp, see PAO1 sequence accession no. AE0040914), and the contained *res* site hunter transposon. For strains AW32, PA8, and PA41, sequences were obtained only for the portion covering tRNA^Lys^-*attL* through to the *orfX* insertions. Symbols: ^1^ clinical isolate; ^2^ non-clinical isolate; ^3^ unknown source; * lacks IS*407*. Distinctive features of the GIs: PAGI-5 [6] has novel regions NRI (orange box) and NRII (blue box), IS (=IS*407*, black box), *orfX* (=*orf30* at nt 27,957–28,256). PAGI-5v and one-step derivatives with an inserted *res* site hunter transposon have IS (=IS*222*, red box), two *orfs* (black circle), *orfX* with inserted Tn*6603* (black arrowhead), *orfX*::Tn*6603* with inserted *res* site hunter (grey arrowhead), NRII* (proximal 6.2 kb of NRII). PAGI-5v multistep Tn*502*-derivative (yellow arrow head) has 1.3 kb novel DNA (grey box; hypothetical protein), all but 278 bp of NRII* is replaced by a 6.5 kb fragment containing phage-related genes (green box). The hypothetical ancestral GI has *orfX* and NRII*. Broken arrows indicate that multiple events have occurred to generate the descendant; solid arrow indicates a single event. Mobility-related functions are located in the following regions: *orf1* integrase and *orf2* MOB_H_-type relaxase in *attL*-NRI; *orf34* MPF_G_-type coupling protein in *orfX*-NRII and *orf80–orf91* pilus proteins in NRII-*attR*. Attachment sites, *attL* and *attR*.

Scrutiny of the GenBank sequences that contained *orfX*::Tn*6603* revealed that this feature was located towards one end of a large DNA segment (ca 94.4 kb), which is a variant of genomic island PAGI-5 (99.4 kb), a member of the ICE family of mobile elements [3,6,64]. Both the variant island, PAGI-5v, and PAGI-5 probably share a common ancestor whose conserved backbone subsequently accumulated different exogeneous genes in novel regions NRI and NRII, as defined by Battle et al. [6] (Figure 4). These regions straddle *orf30*, the *orfX* homologue in PAGI-5. NRI of PAGI-5 and a nearby IS*407* are replaced in PAGI-5v by a simple insertion of IS*222* (flanked by a 3 bp DR, CCC) [65] and a pair of *orfs*. Part of NRII (6.2 kb) is present in both islands, with additional cargo in PAGI-5, including MGE remnants (phage-type genes and Tn*5041*-like *mer* genes). Descendants of the PAGI-5v lineage have acquired a *res* site hunter (forming Tn*6603*::Tn elements) whilst maintaining a relatively unchanged backbone. One exception is the Tn*6603*::Tn*502* strain (Figure 4). It has an extra component in NRI, and all except 278 bp at the start of NRII are replaced by 6.8 kb of novel DNA. The sequence of events responsible for these various differences and the origins of the components are unknown. We obtained DNA sequences for local strains AW32, PA8, and PA41, sufficient to confirm that they too contain the short arm of PAGI-5v (*attL*-Tn*6603*::Tn); we presume the remainder of the island is also present. Collectively, the *P. aeruginosa* strains that have PAGI-5 or PAGI-5v are geographically dispersed, with the island situated adjacent to either of two tRNA^Lys^ genes (Figure 4). The latter feature suggests a capacity for lateral movement, and has been observed experimentally for several members of the pKLC102 family, which includes PAGI-5 [66,67]. Such movement is also supported on functional grounds, since these islands and PAGI-5v (e.g., in ST395, Figure 4) encode an integrase of the DNA_BRE_C-type (Orf1 of [6]) [68]. Moreover, pKLC102, PAGI-5, and PAGI-5v have conjugation-like functions, including a coupling protein (Orf34 of [6]) and relaxase (Orf2 of [6]), which are characteristic of the MPF_G_ and MOB_H_-types, respectively. This integrase–MPF–relaxase combination has been described previously among ICEs [68,69]. Lastly, pilus proteins required for the conjugation mechanism of PAPI-1, and which are related to those of enterobacterial plasmid R64, have homologues in PAGI-5 (Orf80–Orf91 of [6]) and pKLC102 [70], as well as in PAGI-5v. Taken together, these various features highlight evolutionary, often module-based relationships between conjugative plasmids, GIs, and ICEs, as noted by others [69,71,72].

### 3.5. Detection of Tn6606 and Related Elements: Defective Transposons of the Tn5053 Family

**Figure 5:**
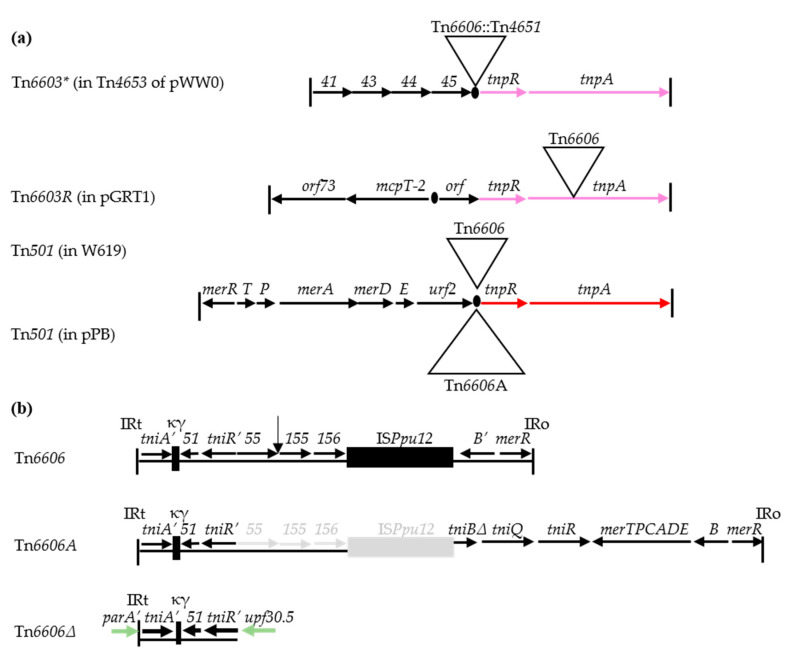
(**a**) Structures of Tn6603*, Tn*6603R,* and Tn*501* with an inserted Tn*6606* family transposon. The genes and *orfs* are represented as named or numbered arrows, the *res* sites as black circles, and the IRs (38 bp) as vertical lines. Transposition modules shown in pink (>99% identical) are less similar to that in red (94% identical). Triangles show the locations of a Tn*6606* family transposon. The structures shown are present in plasmids pWW0, pGRT1, pPB, and the chromosome of strain W619 (see Table 3). The putative products of *orf41*–*orf45* are named in Figure 3a; the *mcpT-2* gene encodes a methyl-accepting chemotaxis protein and *orf73* encodes a hypothetical protein. In all except in pGRT1, the *res* region is split into two parts by the inserted transposon. (**b**) Structures of Tn*6606* family transposons. The positions of IRs (25 bp) are shown and remnant genes are indicated by prime notation (e.g., *tniA’*). Inserted MGEs are represented as boxes, except for Tn*4651,* whose position in Tn*6606* is marked by the vertical arrow. The region shown in grey is hypothetical, as the complete sequence of pPB is unknown. Tn*6606Δ* is present in R772 (IncP-1β), whose sequences are shown in green. The putative products of the *orfs* encode the transcription regulator proteins (Orf51 and Orf55), putative drug resistance protein (Orf155), and putative multidrug exporter (Orf156). The figure was generated from the following GenBank entries: pWW0 (NC_003350); pGRT1 (HM626202); W619 (CP000949); pPB (U90263, U81032 and U80214); R772 (KF743817).

Apart from the Tn*6603* and Tn*6603*-Tn sequences found in GenBank, we discovered two variants of Tn*6603*. One, named Tn6603* (6693 bp), lacks the *res*-associated 465 bp *orf* (Figure 3); it forms the base element of a complex transposon (Tn*4653*) in *P. putida* plasmid pWW0 [63] (Figure 5a). The other is a recombinant element, named Tn*6603R* (7672 bp). It contains a *tnp_6603_* module associated with a different cargo module, and forms one end of a Tn*4653*-like transposon in *P. putida* plasmid pGRT1 [73]. Tn6603* and Tn*6603R* were of particular interest because their sequences are interrupted by the same *mer-/tni*-defective *res* site hunter. The defective element, named Tn*6606*, appears in different guises in the database, forming a family of elements. These are Tn*6606* (in pGRT1 and the chromosome of *P. putida* W619); Tn*6606*::Tn*4651* (in pWW0, an IncP-9 plasmid); Tn*6606A*, a large, possibly ancestral element present in *P. stutzeri* plasmid pPB; and Tn*6606Δ*, a remnant present in R772 (IncP-1β) from a clinical *Proteus* strain (Figure 5b; Table 3).

Tn*6606* displays features characteristic of the Tn*5053* family. It is bordered by 25 bp IRs, which enclose remnant modules *tniA’ tniR’,* and *merR merB’* (Figure 1 and Figure 5b). The entire IRt*-tniA’* sequence (522 bp) and the *tniR’* remnant (663 bp) are most similar to those of the prototype Tn*5053* (99% and 73% identical, respectively). The sequence discrepancy, however, and the orientation of *tniR’* opposite to that found typically (Figure 1) suggests that the IRt*-tniA’* and *tniR’* portions were derived from different Tn*5053*-like elements. The remnant *mer* module aligns with part of an organomercurial resistance module in pPB (99% identity), hence the suggestion that Tn*6606A* in pPB may be ancestral to Tn*6606*. The other components of Tn*6606* also suggest a complex evolutionary history. They include “orphan” genes (*orf51-55-155-156*; Figure 5b; [63], κϒ [56] and the transposable IS*Ppu12* [78]). None of the components of Tn*6606* are associated with a known phenotype.

Features at the target site of each of the Tn*6606* family members is indicative of transpositional activity. First, although these elements lack a *tni* module, all except Tn*6606Δ* retain both IR borders, which are flanked by 5 bp DRs. Second, each element is inserted within or close to a *res* site (i.e., in resI-resII_6603*_ for Tn*6606*::Tn*4651* (Figure 3); in the same *resII_501_* site for Tn*6606* and Tn*6606A*; in *tnpA_6603R_* for Tn*6606*; and near *res_IncP_* for Tn*6606Δ*). Third, each element is in the typical orientation adopted by *res* site hunter elements (i.e., IRo adjacent to *tnpR*). We conclude that the transposition of these elements to the observed sites was facilitated by *tni* functions provided in trans. These events occurred on at least four separate occasions involving three different *re*s-target regions: *res_6603_*-like, supporting observations made earlier in this report; *res_501_*, identifying this as an alternative target site for Tn*5053* family transposons; and *res_IncP_*, a known target for Tn*5053*/Tn*402* members generally.

The presence of Tn*6606Δ* in *res_IncP_* of R772 (IncP-1β) is of interest, as it is the sole example of a Tn*6606* relative outside the *Pseudomonas* genus and in a clinically sourced strain (Table 3). It is possible that an intact Tn*6606* originally targeted *parA-res_IncP_* of an R772 ancestor, and via *tni*-assisted transposition miss-inserted into *parA* (a 264 bp remnant of *parA* remains; Figure 5b). Subsequent recombination events, either spontaneous or caused by the activity of Tn*6606* or its IS*Ppu12* component, resulted in deletion of Tn*6606* and R772 sequences, leaving the extant structure (Figure 5b). Such losses of DNA, especially those promulgated by accrued MGEs, have been proposed to account for genetic variability, which is particularly evident in this region of IncP-1β plasmids [11,79].

Collectively, these findings point to encounters in non-clinical environments that have enabled *tni*-functional elements to promote the lateral movement of *tni*-defective elements between MGEs. The possible participants, in this case IncP-1 plasmids, IncP-9 plasmids, and *mer* transposons, have been found to cohabit environments polluted by man-made or natural contaminants [80,81].

## 4. Discussion

The main results from this study are that (i) members of the Tn*5053* family of *res* site hunter elements are present in *P. aeruginosa* clinical strains; (ii) these *res* site hunters form nested associations predominantly with Tn*6603*, a novel carrier transposon; (iii) a historical event that linked Tn*6603* with a PAGI-5-related genomic island preceded acquisition of a *res* site hunter on at least five subsequent occasions.

We isolated five types of Tn*5053* family transposons from local hospital strains of *P. aeruginosa* (Tn*502*, Tn*512*, Tn*5053*, Tn*5058v2,* and the new element Tn*6604*) (Table 2, Figure 1). This substantial cohort included elements from 30-year-old strains, suggesting a long-standing association with the local clinical setting. All of the identified transposons had functional *mer* and *tni* modules and were chromosomally located. In most cases, this was a consequence of typical *res* site hunting activity directed to a carrier transposon (discussed below). However, in four strains with Tn*502* or Tn*512*, the chromosomal locations were not linked to obvious *res* or resolvase gene sequences (Table 2, footnote j). In one case, Tn*502* was flanked by DRs, consistent with its insertion by transposition. A similar preservation of DRs also attends chromosomal insertions of Tn*5053* to non-*res* sites under laboratory conditions [19]. In the other three cases, DRs were absent; these were possibly deleted due to transposon activity following the insertion event, as observed with Tn*502* [37]. Chromosomally inserted *mer*-type *res* site hunters in each of these two categories were found in GenBank sequences of clinical and non-clinical strains. Therefore, although *res* site hunters transpose only rarely in the absence of an external resolvase, such events occur in nature and may contribute to the spread of these elements more than is appreciated.

Chromosomally located Tn*6603* was identified as a significant carrier of *mer*-type *res* site hunters. It was detected in the surveyed strains in its “unloaded” and nested forms (6 and 7 examples, respectively; Table 2) and in GenBank sequences (14 and 10 examples, respectively) (Table S1). *P. aeruginosa* clinical strains served almost exclusively as the hosts. In about half of the strains, the transposon was located at disparate chromosomal sites, consistent with the low-level target specificity exhibited experimentally by Tn*6603c*. In the remaining strains, Tn*6603* (or the nested element) was located in PAGI-5v, a relative of genomic island PAGI-5 in the ICE family [3,6,64]. The PAGI-5v lineage was comprised of an ancestral *P. aeruginosa orfX*::Tn*6603* strain and derivatives that later acquired a *mer*-type *res* site hunter. Strains with PAGI-5v- or PAGI-5-type islands are globally distributed, with the oldest two isolated in the 1990s (Figure 4). Some specific sequence groups are widespread (ST395, ST155, and ST27) and the islands are sited at one or other of the two chromosomal tRNA^Lys^ gene copies (Figure 4; [82]). This distribution suggests a capacity for lateral movement between bacteria and between integration sites, as has been demonstrated for members of the pKLC102 family, which includes PAGI-5/PAGI-5v [66,67,82]. The different locations of the *res* site hunter in *res_6603_* of PAGI-5v show that Tn*5053* and Tn*502* were inherited on several separate occasions, and Tn*6604* was inherited once (strain PA41 is the sole example) (Table 2; Figure 3). Tn*5053* occupies either of three sites in the *resI*-*resII* region of *res_6603_*, similar to its distribution in *res* of Tn*1721* [19]. The middle site appears to be favored, however the nine strains have near-identical *orfX*::Tn*6603*::Tn*5053* sequences and four are ST155 strains. Therefore, all nine may derive from a single Tn*5053* acquisition event. In contrast, the eight examples of Tn*502* in *resII* may identify an actual favored site, since most were independent transposition events (five isolated in this study and at least one of the remaining examples). The different targets selected by Tn*5053* and Tn*502* may reflect subtle differences in their transposition processes, similar to those demonstrated between Tn*502* and Tn*512* [37]. These data in toto identify a minimum of five occasions on which an *orfX*::Tn*6603* strain acquired a *res* site hunter. The nested element in strain RH19 is not in *orfX* (Table 2); it may represent a sixth acquisition event or merely transposition of the element from a pre-existing *orfX*::Tn*6603*::Tn*502* lineage. Whether these interactions occurred inside or outside the clinical setting and whether the first involved Tn*6603* in PAGI-5v or was separate from it is not known.

Five instances of plasmid-associated Tn*6603* were detected in GenBank sequences. Four involved catabolic plasmids in isolates from non-clinical sources (natural or polluted soils). Tn*6603* was present in *P. fluorescens* plasmid pG69 (from Estonia; accession no. MH061177) and *P. taiwanensis* plasmid pSTY (from Germany; accession no. CP003962), whereas a minor variant, Tn6603*, occurred in pWW0 [63], while a hybrid, Tn*6603R*, which has different cargo genes, occurred in pGRT1 [73] (Figure 5a). Only pWW0 and pGRT1 have been characterised and both were from *P. putida* strains (from Japan and Belgium, respectively). These examples imply that reticulated transmission of Tn*6603* occurs in nature, sometimes leading to structural changes in the transposon. Such changes were not present amongst the clinical isolates, perhaps indicating more recent occupancy in *P. aeruginosa*. The connection with catabolic plasmids also raises the possibility that the gene cargo in Tn*6603*, which has no demonstrated function, may provide an adaptive role in non-clinical environments. Although the catabolic plasmids are large (117–321 kb) and encode conjugation-like genes, only pWW0 and pGRT1 have demonstrated conjugal ability and pWW0 is in the IncP-9 group. IncP-9 plasmids have a relatively limited host range, however they are stable in several *Pseudomonas* species, including *P. aeruginosa* [63,83], and can deliver transposons across generic boundaries [84]. Therefore, transfer pathways for Tn*6603* family elements from environmental *Pseudomonas* spp. to clinically significant *P. aeruginosa* strains can be envisaged. One example of plasmid-associated Tn*6603* in *P. aeruginosa* has been reported (accession no. CP027175). Although the source of the strain was not specified, the plasmid (72 kb) encodes conjugation-like genes, therefore the potential for lateral transmission within this species already exists.

The Tn*6603* variant that occurs in pGRT1 is nested with Tn*6606*, a *mer-*/*tni*-defective transposon that groups with several related elements (Figure 5b). A different defective transposon, also in the Tn*5053* family, has been described in an IncP-1ϒ plasmid from river epilithon [24]. Named here as Tn*6607*, it retains *mer_5053_* and intact *res-tniR_5053_* components, but is otherwise *tni*-defective (Figure 1). These various defective elements are all located in or near a *res* site (*res_6603_*, *res_501_*, r*es_IncP__β_*, or *res_IncP__ϒ_*) and the four that retain IR termini are flanked by 5 bp DRs. Therefore, dependence on another element to provide *trans*-acting *tni* functions operates successfully in nature to relocate defective transposons. Similar assisted relocations of class 1 integrons are identifiable among clinical strains [15,40]. These defective transposons typically lack the native *res* site, as well as *tni* functions, but this does not undermine cointegrate resolution, as the target site resolvase aids resolution via the host RecA system [37]. On the other hand, transposition to a site lacking an external resolvase yields long-lived cointegrates because the RecA-mediated process is inefficient. The *mer*-type defective elements mentioned here have complex mosaic structures, suggesting successive recombination and recruitment events. They also encode *orfs* that may be connected to catabolic functions, perhaps relevant to strain adaptation in natural environments. We are not aware of other defective members of the Tn*5053* family.

Tn*501* and Tn*1013^#^* were identified as carriers of *mer*-type *res* site hunters, although examples were limited to Tn*501*::Tn*6606* elements (Figure 5a), Tn*1013^#^*::Tn*5058v2* (Table 2), and a Tn*1013*::Tn*512* sequence from a *P. alkylphenolica* soil isolate (accession no. CP009048). Tn*501* is common in various GenBank sequences, whereas Tn*1013* appeared in *P. aeruginosa* strains, occurring in an environmental IncP-1α plasmid [54] and in plasmid and chromosomal sites in clinical strains (accession no. MN433457 and CP031677). These few associations with *mer*-type *res* site hunters are nevertheless consistent with reports of class 1 integrons in Tn*1013*, its relative Tn*1403*, and in Tn*5051* and Tn*1721*, which have links with Tn*501* [23,41,58]. These carriers and Tn*6603* form two distinguishable groups based on the similarity of their transposition (*tnp*) modules (*tnp_501_* vs. *tnp_6603_* (94.1%); *tnp_1013_* vs. *tnp_1403_* (94.6%); *tnp_1013_* vs. *tnp_6603_* (91.9%)) and alignment differences in a 32 bp segment that abuts the *tnpR* start codon [59]. Thus, Tn*1013* (and Tn*1013^#^* from this study) groups with Tn*1403,* whereas Tn*501* groups with Tn6603*, the base element of Tn*4653* (Figure 5a). By extension, Tn*6603* (and Tn*6603R*) belongs to the latter group, even though its 32 bp segment is replaced by a 465 bp *orf* (Figure 3). Apart from *orf_465_*, Tn*6603* and Tn6603* have identical *tnp* and cargo modules. A different cargo module is common to Tn*1013* and Tn*1403* (Figure 3; [59]). These various distinguishing features identify potential evolutionary lineages within a broadly related group of elements in the Tn*21*/Tn*501* group.

Our experimental focus was on transposon targets of *mer*-type *res* site hunters in clinical strains. Although these had not previously been defined, possible targets were known from laboratory studies using Tn*5053* [19] and the natural targets of Tn*402* family members [41,58]. We can now add to the limited list of *mer*-sensitive transposon targets a third example involving a Tn*1013*-like element (Tn*1013^#^*) and the new element, Tn*6603*, which predominated in our strains. Tn*6603* was conspicuous because of its strong association with *P. aeruginosa* clinical strains beyond those tested, and because of its occurrence in PAGI-5v, an ICE whose relatives in the pKLC102 family are abundant in *P. aeruginosa* and can contribute to virulence [6,82,85]. Genomic islands, including examples in the ICE group, also have proposed roles in the prevalence and transfer of antimicrobial resistance in *P. aeruginosa*, often through their carriage of class 1 integrons, which may be embedded in *mer*-sensitive or *mer*-resistant transposon carriers [17,38,61,62,64,86]. The presence of Tn*6603* in PAGI-5v, or separately in the chromosome, raises the prospect of future acquisition of class 1 integrons and their dispersal via the GI/ICE or via another MGE that acquires the Tn*6603*::In element or In alone. The *mer*-sensitive Tn*4661* transposon, unrelated to Tn*6603*, is implicated in similar interconnected transmission routes. Tn*4661* is part of PAGI-4 in *P. aeruginosa* C strains [87] and was found to be plasmid-borne, with an added nested integron (Tn*6061*), among other clinical isolates in which Tn*6061* was chromosomal [62]. Class 1 integrons, although not self-mobile, can independently relocate using *tni* functions supplied in trans. The *mer*-type *res* site hunters that cohabit with integrons can provide these functions, either from a chromosomal location, as we found, or from a plasmid (e.g., IncP-1α plasmids; [54,88]). Functional Tn*402* transposons are an alternative source. We note with interest a recent example of a Tn*6603*::Tn*402* element encoding an *oxa2* gene cassette in a *P. aeruginosa* clinical strain from Colombia (accession no. CP029605). The strain belongs to the globally dispersed ST235 clone and our analysis identified Tn*6603*::Tn*402* within *orfX* in PAGI-5v (Figure 4). We anticipate the emergence of other examples that link Tn*402* family members, both the fully functional and class 1 integron types, to PAGI-5v through *res*-targeting of Tn*6603*.

## Figures and Tables

**Table 1:** Bacterial plasmids used.

Plasmid ^a,b^	Relevant Features or Derivations	Reference/Source
**Conjugative Plasmids**		
pUB307	Ap^s^ deletant of RP1; has *parA-res_IncP_* region; Tc^r^ Km^r^	[48]
pUB1601	*Pst*I-ligation derivative of RP1; lacks *parA-res_IncP_* region; Tc^r^ Km^r^	[49]
pSP116 ^c^	pUB1601*tet*::Tn*6603c*; Km^r^ Cm^r^	This work
pSP144	pUB1601*tet*::Ω1Tn*502*; Km^r^ Hg^r^	[37]
pSP145	pUB1601*tet*::Tn*501*; Km^r^ Hg^r^	This work
**Nonconjugative Plasmids**		
pBluescript II SK+ (pSK)	*E. coli* vector; Ap^r^	Stratagene
pACYC184	*E. coli* vector; Cm^r^ Tc^r^	[50]
pBR322	*E. coli* vector; Ap^r^ Tc^r^	[51]
pVS76 (pBR322*-res_IncP_*)	pBR322 with *bla* disrupted by a clone of the RP1 *parA-res_IncP_* region; Tc^r^	[52]
pVS982	pBR322::Tn*501*; Ap^r^ Tc^r^ Hg^r^	[53]
pVS983	pBR322*tet*::Tn*502*; Ap^r^ Hg^r^	[53]
pSP111 ^d^ (pSK-Tn*6603*)	pSK with a clone of Tn*6603* from *P. aeruginosa* PA65; Ap^r^	This work
pSP112 ^e^ (pSK-Tn*6603c*)	pSP111 with a clone of the *cat* gene from pACYC184; Ap^r^ Cm^r^	This work
pSP114 ^f^ (pBR322*-res_6603_*)	pVS76 with *parA-res_IncP_* sequences removed and replaced with *tnpA-orf-res* region of Tn*6603*; Tc^r^	This work

^a^ Additional transpositional derivatives of pUB307 and pUB1601 are listed in Table 2. ^b^ Synonyms are shown in parentheses and are used in the text for simplicity (e.g., pSP111 = pSK-Tn*6603*). ^c^ A DH5α derivative carrying pSP111 and pUB1601 was mated with PAO9503 (selection was on NA.Sm.Cb). A mixture of transconjugants was subcultured on the same medium then plasmid DNA was extracted and transformed into DH5α, with selection on NA.Km and subsequent screening to identify Tc^s^Cb^s^ transformants. Restriction enzyme-profiling of plasmid DNA and subsequent sequencing was performed to obtain pUB1601*tet*::Tn*6603*. ^d^ Primers used for PCR amplification of a 7439 bp region containing Tn*6603* from the chromosome of *P. aeruginosa* PA65 were VR56 (5′-GCGGGATCCTGATCGAGATTGCGTCAGC) and VR67 (5′-GCGCTCGAGGATGTATCGAATCCGGCTC), containing *Bam*HI and *Xho*I sites (underlined) at the ends, which were used for cloning purposes. ^e^ Primers used for PCR amplification of a 1380 bp region containing the *cat* gene from pACYC184 were VR75 (5′-GCGAGATCTCATCTGTATTAACGAAGCGC) and VR76 (5′-GCGACGCGTAGTGCCAACATAGTAAG CC), which contain at one end a *Bgl*II restriction site and at the other a *Mlu*I restriction site; the sites (underlined) were used for cloning into *Mlu*I/*BgI*II-digested pSP111 (see Figure 3). f The pVS76 *Xho*I-*Mlu*I-fragment (5810 bp) was ligated with the *Xho*I-*Mlu*I 4992 bp fragment of Tn*6603* from pSP112. The ligation replaces RP1 *parA-resIncP* sequences with Tn*6603* sequences from IRt-*orf45′* (see Figure 3). Abbreviations: resolution site, *res*; partition gene, *par*; tetracycline, chloramphenicol, beta lactamase resistance genes, *tet, cat, bla* respectively; transposase gene, *tnp*; see Section 2.1 for antibiotic abbreviations.

**Table 2:** Identification of Tn*5053* family transposons and their carrier transposons in mercury(II)-resistant clinical strains of *P. aeruginosa.*

*P. aeruginosa* Strain	Year Isolated ^a^	Transfer Frequency to pUB307; Tn Detected ^f,g^	Transfer Frequency to pUB1601; Tn Detected ^f,h,i^	Location of Nested Tn (or Tn) in Clinical Strain ^j^
RH19	1992 ^b^	6.2 × 10^−3^; Tn*502*	1.6 × 10^−6^; Tn*6603*::Tn*502*	Ω1
AW32	1991 ^c^	7.2 × 10^−3^; Tn*502*	3.2 × 10^−8^; Tn*6603*::Tn*502*	*orfX*
AW57	1991 ^c^	1.1 × 10^−3^; Tn*502*	1.2 × 10^−8^; Tn*502*	Ω2
PA9	2015 ^d^	2.8 × 10^−2^; Tn*502*	2.0 × 10^−8^; Tn*502*	Ω3
AW54b	1991 ^c^	2.5 × 10^−4^; Tn*512*	<1.0 × 10^−8^; ND	Ω4
AW60	1991 ^c^	1.6 × 10^−4^; Tn*512*	<1.0 × 10^−8^; ND	Ω4
PA8	2015 ^d^	1.6 × 10^−4^; Tn*5053*	1.0 × 10^−5^; Tn*6603*::Tn*5053*	*orfX*
PS1	1988 ^e^	0.5 × 10^−4^; Tn*5053*	7.0 × 10^−6^; Tn*6603*::Tn*5053*	*orfX*
AB1	1992 ^c^	1.2 × 10^−4^; Tn*5053*	3.4 × 10^−6^; Tn*6603*::Tn*5053*	*orfX*
AW1	1991 ^c^	0.6 × 10^−4^; Tn*5053*	1.0 × 10^−5^; Tn*6603*::Tn*5053*	*orfX*
PA118	2015 ^d^	1.2 × 10^−2^; Tn*5058v2*	1.2 × 10^−6^; Tn*1013^#^*::Tn*5058v2*	Ω5
PA41	2015 ^d^	2.9 × 10^−5^; Tn*6604*	3.0 × 10^−7^; Tn*6603*::Tn*6604*	*orfX*

^a^ The strains listed hybridized to a *tniA_502_* probe. Two others had a truncated *tniA* gene and were Hg(II)-sensitive; they were not studied further. None of the listed strains produced transconjugants on NA.Sm.Hg (<10^−8^/donor) when mated with 43 °C-grown PAO9503. ^b,c,d,e^ Indicating clinical source of the strains. ^f^ Represents the recovery of Hg(II)-resistant transconjugants per donor. The clinical strains carried pUB307 or pUB1601; the recipient was PAO9503. ^g^ The transposons were inserted in the pUB307 *res* region within position 35,014–35,020 (accession no. BN000925) in *resII*. All are 99–100% identical to the previously named prototypes, except for the new transposon, Tn*6604*, and Tn*5058v2* (see text). ^h^ The nested transposons were inserted in pUB1601 at positions near *oriV, klaC, klcB, klaB,* and *korC*. In matings involving AW57 and PA9 donors, Tn*502* alone transposed to pUB1601. ^i^ The Tn*6603* sequences were identical, except for a single base-pair difference in Tn*6603* from strain PA8. Tn*1013*^#^ from strain PA118 is related to the prototype Tn*1013* [54] (see text). ^j^ Tn*6603*-nested elements were inserted in *orfX* (see text), except for in strain RH19. Symbols Ω1–Ω5 indicate insertions at different chromosomal sites. The latter were determined from contigs that included the *res* site hunter (Tn*502* or Tn*512*) or nested element (Tn*6603*::Tn*502* or Tn*1013^#^*::Tn*5058v2*) and 3.0–7.0 kb of chromosomal DNA on each flank. DRs adjoined Tn*6603* in Ω1 (CACAT), Tn*502* in Ω2 (TAATT), and Tn*1013^#^* in Ω5 (TGAAT). The Ω1–Ω4 insertion sites were within hypothetical genes or between such genes, except for Ω5, which was within an *orf* encoding a membrane fusion protein efflux pump. ND = not detected.

**Table 3:** Bacterial strains that carry Tn*6606* or related elements.

Bacterium	Source (Decade)	Transposon and Carrier	Ref
*Pseudomonas putida* mt-2	Field soil/Japan (1960)	Tn*6606*::Tn*4651* in pWW0 (IncP-9)	[74]
*P. putida* DOT-T1E	Wastewater/Spain (1990)	Tn6606 in pGRT1 ^a^	[73]
*P. putida* W619	Poplar root/stem/Belgium (2000) ^b^	Tn6606 in chromosome	[75]
*Pseudomonas stutzeri* OX	Wastewater/Italy (1980)	Tn*6606A* in pPB ^a^	[76]
*Proteus mirabilis*	Clinical isolate/USA (1970)	Tn*6606Δ* in R772 (IncP-1β)	[77]

^a^ Unknown incompatibility type. ^b^ Personal Communication from Dr. D. van der Lelie.

## Supplementary Materials

The following are available online at https://www.mdpi.com/2076-2607/8/12/1997/s1: Table S1: Strains containing Tn*6603* or Tn*6603*::Tn in GenBank.
