# Supplementary material for: Tn6603, a Carrier of Tn5053 Family Transposons, Occurs in the Chromosome and in a Genomic Island of Pseudomonas aeruginosa Clinical Strains
Source: Microorganisms. 2020 Dec 15;8(12):1997. doi: 10.3390/microorganisms8121997 (PMC7765201; doi:10.3390/microorganisms8121997)
Supplement: Supplementary file 1 [file microorganisms-08-01997-s001.pdf]

Table S1 Strains containing Tn*6603* or Tn*6603*::Tn in GenBank.

Accession No.	*P. aeruginosa* strain	ST	DRs flanking Tn*6603* or Tn*6603*::Tn	Insertion location of Tn*6603* or Tn*6603*::Tn[a]	Year[b]/Country	Source	*res* hunter	DRs flanking *res* hunter	Insertion site (nt) in Tn*6603*[c]
CP033832	FDAARGOS_505	179	ATCTA	*orf1*	2015/USA	Tracheal aspirate	-	-	-
CP033684	H26027	17	ATCTA	*orf1*	2010/Switzerland	Human Skin	-	-	-
CP008861	H47921	1105	ATCTA	*orf1*	2016*/USA	Clinical	-	-	-
CP006931	SCV20265	299	ATCTA	*orf1*	2013*/Germany	Human	-	-	-
CP025229	AK6U	179	AGGAA	*orf2*	2012/Kuwait	Soil	-	-	-
CP025229	AK6U	17	No DRs	*orf3*	2012/Kuwait	Soil	-	-	-
CP027175	AR_0230; Unnamed plasmid	NA	No DRs	*orf4*	2018*/USA	N/D	-	-	-
MH061177	*P. fluorescens* strain P69 plasmid pG69,	NA	CTGAG	*orf5*	2018*/Estonia	Oil polluted area	-	-	-
CP003962	*P. taiwanensis* VLB120 plasmid pSTY	NA	TAGAC	*orf6*	2014*/Germany	Soil	-	-	-
LR134308	NCTC11445	N/D	ACCAA	*orfX*	2018*/UK	N/D	-	-	-
CP029090	AR442	395	ACCAA	*orfX*	2018*/USA	N/D	-	-	-
LT969520	RW109	111	ACCAA	*orfX*	2017*/UK	N/D	-	-	-
CP013993	DHS01	395	ACCAA	*orfX*	1997/France	Patient nose	-	-	-
CP023255	CCUG 70744	395	ACCAA	*orfX*	2013/Sweden	Sputum	-	-	-
CP039749	PRD-10	252	TCTAC	Intergenic	2019*/USA	Water bottle	Tn*5053*	TCTAT	4313
CP041013	FDAARGOS_610	252	TCTAC	Intergenic	2019*/USA	Water bottle	Tn*5053*	TCTAT	4313
LR590473	NCTC13359	252	TCTAC	Intergenic	2019*/UK	Water bottle	Tn*5053*	TCTAT	4313
CP015117	ATCC 27853.1	155	TGCTC	*orf7*	2014/Netherlands	Human	Tn*5053*	GCGCG	4330
CP015117	ATCC 27853.2	155	ACCAA	*orfX*	2014/Netherlands	Human	Tn*5053*	GCGCG	4330
LR134309	NCTC12903	155	ACCAA	*orfX*	2018*/UK	Blood culture	Tn*5053*	GCGCG	4330
CP011857	ATCC 27853	155	ACCAA	*orfX*	2015*/Hong Kong	Sputum	Tn*5053*	GCGCG	4330
CP022001	Pa1207	155	ACCAA	*orfX*	2012/Mexico	Blood Human	Tn*5053*	GCGCG	4330
CP030328	AR_455	298	ACCAA	*orfX*	2018*/USA	NA	Tn*502*	ATGTG	4288
CP008864	W60856	959	ACCAA	*orfX*	2014*/USA	Clinical	Tn*5053*	GTTCT	4335

[a] *orf*'s encode hypothetical proteins with no similarity to proteins of known function except *orf7* that shares homology with a M48 family metalloprotease. [b] Year isolated / * Year submitted to GenBank database as isolation date was not available. [c] Coordinate according to Tn*6603* GenBank Accession No. MT043136, ST-Sequence type. N/D-No data; NA–Not Applicable; ST- Sequence type. Transposons detected were >99% identical.
